# The efficacy of meditation on insula dysfunction in patients with chronic schizophrenia

**DOI:** 10.3389/fpsyt.2025.1622594

**Published:** 2025-09-02

**Authors:** Di Kong, Shoukang Zou, Yanqing Huang, Zezhi Li, Mi Yang, Donghong Cui

**Affiliations:** ^1^ Department of Forensic Expertise, The Fourth People’s Hospital of Chengdu, Chengdu, China; ^2^ Unit 4—Department of Geriatric Medicine, The Fourth People’s Hospital of Chengdu, Chengdu, China; ^3^ School of Life Science and Technology, University of Electronic Science and Technology of China, Chengdu, China; ^4^ Department of Nutritional and Metabolic Psychiatry, The Affiliated Brain Hospital, Guangzhou Medical University, Guangzhou, China; ^5^ Department of Psychiatry, Guangdong Engineering Technology Research Center for Translational Medicine of Mental Disorders, Guangzhou, China; ^6^ Key Laboratory of Neurogenetics and Channelopathies of Guangdong Province and the Ministry of Education of China, Guangzhou Medical University, Guangzhou, China; ^7^ Department of Science and Education, The Fourth People’s Hospital of Chengdu, Chengdu, China; ^8^ The Clinical Hospital of Chengdu Brain Science Institute, MOE Key Lab for Neuroinformation, University of Electronic Science and Technology of China, Chengdu, Sichuan, China; ^9^ Shanghai Mental Health Center, Shanghai Jiao Tong University School of Medicine, Shanghai, China

**Keywords:** schizophrenia, meditation, fMRI, insula, dynamic functional connection

## Abstract

**Objectives:**

Meditation combined with antipsychotic medication can effectively improve the clinical symptoms and prognosis of patients with schizophrenia, but its underlying neural circuit mechanism is not clear. Many previous studies have shown that the symptoms of schizophrenia are related to dysfunction of the insula. We aimed to explore the neural circuitry mechanisms associated with insular dynamic functional connectivity in the treatment of schizophrenia with meditation combined with antipsychotics in a prospective controlled study.

**Methods:**

30 chronic schizophrenia patients were accepted meditation + antipsychotic drug intervention for 8 months. At baseline, 29 age-matched normal healthy controls were used to identify the differential brain regions of dynamic functional connectivity between the insula subregions and the whole brain in the schizophrenia patient group. PANSS scale scores, RBANS scale scores and MRI data were collected at baseline, third month, and eighth month in the schizophrenia group, MRI data was collected at baseline in the healthy control group, then dynamic functional connectivity analysis of the whole brain was conducted using the six subregions of the insula(the left ventral anterior insula (L-vAI), right ventral anterior insula (R-vAI), left dorsal anterior insula (L-dAI), right dorsal anterior insula (R-dAI), left posterior insula (L-PI), and right posterior insula (R-PI)) as seed. Identify which dynamic functional connections in the differential brain regions were improved after meditation intervention at third and eighth month compared with baseline.

**Results:**

At baseline, global functional connectivity was significantly lower in the meditation group than in the healthy control group in the left orbital inferior frontal gyrus, right orbital inferior frontal gyrus, medial and paracingulate gyri, right hippocampus, and left auxiliary motor area. At the third and eighth month, schizophrenia patients in the meditation group showed significant improvement of functional connectivity between L-dAI、L-PI and right orbital part of inferior frontal gyrus compared with baseline. Although the PANSS scale scores were significant improvement in the meditation group at the third and eighth month than baseline, there was no significant difference in the RBANS scale scores.

**Conclusion:**

Long-term treatment with meditation can improve the overall psychiatric symptoms of patients with schizophrenia and the abnormal dynamic functional association of the insula, which provides a clinical and neuroimaging basis for the widespread application of meditation in the treatment of schizophrenia.

## Introduction

Schizophrenia is mainly manifested by clinical psychotic symptoms, which are often accompanied by a decline in social function and impaired cognitive function. It mostly occurs in young and middle-aged adults, with a lifetime prevalence rate of approximately 1% (1). It is characterized by a high recurrence and disability rate, and some patients tend to develop chronic conditions, manifested by persistent negative symptoms, emotional symptoms, and impaired cognitive function (2, 3). Some studies have demonstrated that schizophrenia is a polygenic disease accompanied by significant brain abnormalities, the brain abnormalities may be related to the clinical manifestations and the choice of antipsychotic drugs (1, 4).

Studies based on structure MRI revealed brain abnormalities of patients with schizophrenia, including the enlargement of lateral ventricles, the reduction in brain volume, particularly in the gray matter of the brain, and the thinning of frontal lobe, temporal lobe and hippocampal cortex (5–7). In functional MRI studies of the brain, some research found that positive and negative symptoms of schizophrenia were associated with abnormal connections between the salience network (SN) and the default mode network (DMN) (8, 9). One study found an overall decrease in insular functional connections and a decrease in the differentiation of the connection spectrum between insular subregions in patients with schizophrenia (10). One meta-analysis suggested that schizophrenic patients have specific substantial dysconnectivity in the insula, lateral posterior central cortex, striatum, and thalamus, which may be characteristic brain biomarkers of schizophrenia (11). In recent years, the dysconnection hypothesis of schizophrenia has received increasing attention (12, 13). A study from Harvard Medical School showed that after 1.5 years of follow-up, patients with first episode schizophrenia had a progressive decrease in bilateral insular gray matter volume compared with patients with first episode affective psychosis and healthy controls (14). Reduced dorsal anterior insula functional connections were associated with impaired cognitive function, whereas enhanced functional connections between the ventral anterior insula and superior temporal sulcus were associated with negative symptoms (15). Based on previous studies, abnormalities in the structure and function of the brain, especially the insula, are clearly associated with schizophrenia.

In recent years, owing to its ability to improve executive function, emotional regulation, and working memory, meditation has gradually become an effective treatment for mental diseases (16, 17). Mindfulness is a central element of various forms of meditation and consists of two complementary components: (1) maintaining a focus on the immediate experience and (2) maintaining an attitude of acceptance toward that experience (18). One 2-year follow-up study revealed that patients with schizophrenia in the mindful psychoeducation group experienced significantly greater improvements in psychiatric symptoms, psychosocial function, insight into disease/treatment, and readmission time (19). After mindfulness practice, enhanced neural connectivity and activation in the left hippocampus, dorsal anterior insula, posterior cingulate cortex, and cerebellum/brainstem can promote cortical arousal and the regulation of functional activities in emotional, conscious, and attentional responses, thereby regulating mood and improving cognition (20). The precise cerebral mechanisms underpinning the use of meditation in the treatment of schizophrenia remain to be elucidated. This uncertainty is a significant impediment to the broader implementation of meditation as a therapeutic intervention for individuals diagnosed with schizophrenia.

Therefore, we aimed to explore the correlations between improvements in psychotic symptoms, cognitive function and insula function in patients receiving meditation training on the basis of conventional drug treatment intervention to provide more evidence for improvements in symptoms related to schizophrenia and clarify the possible mechanism of improvements in insula neuroplasticity.

## Methods

### Participants

In 2018, a total of 315 Han patients with schizophrenia were screened at the Shanghai First Civil Affairs Mental Health Centre. The inclusion and exclusion criteria are outlined below. The inclusion criteria were as follows: (1) aged ≥ 18 years; (2) diagnosis of schizophrenia in accordance with the Diagnostic and Statistical Manual of Mental Disorders, 4th Edition (DSM-IV); and (3) illness duration of ≥ 5 years. (4) the Mini-Mental State Examination (MMSE) score >=24. (5) the subjects exhibited residual symptoms of hallucinations or delusions, as indicated by a positive and negative syndrome scale (PANSS), P3 score of 5 or greater, or a minimum of two scores of 5 or greater on the PANSS P1, P5, P6, G1, G3, and G9 items despite adequate treatment with at least two different antipsychotics. The exclusion criteria were as follows: (1) other serious mental illnesses, such as bipolar disorder, autism, etc; (2) organic brain disease or serious physical disease; and (3) a history of drug dependence/abuse.

Following the screening procedures, 30 eligible patients received meditation therapy in addition to their usual rehabilitation treatment. Throughout treatment, all patients were asked to continue taking the antipsychotic medication previously prescribed by their doctor. We also included a control group of 29 neurologically and mentally healthy individuals as the healthy control group. They were used to identify the differential brain regions of dynamic functional connectivity between the insula subregions and the whole brain in the schizophrenia patient group.

### Protocol

At baseline, the differential brain regions of dynamic functional connectivity between the insula subregions and the whole brain in the schizophrenia patient group was confirmed. Subsequently 30 chronic schizophrenia patients were accepted meditation + antipsychotic drug intervention for 8 months. PANSS scale scores, RBANS scale scores and MRI data were collected at baseline, third month, and eighth month in the schizophrenia group, MRI data was collected at baseline in the healthy control group. Identify which dynamic functional connections in the differential brain regions were improved after meditation intervention at third and eighth month compared with baseline finally.

The protocol was approved by the institutional review committee of the Shanghai Mental Health Center and the first Civil Affairs mental health center. Written informed consent was obtained from all the subjects prior to their participation in the study. All procedures performed followed the ethical standards of the institutional and/or national research committee and the Helsinki Declaration and its later amendments or comparable ethical standards. This eight-month controlled trial was registered in the Chinese Clinical Trial Registry (ChiCTR1800014913; Registration Date: 2018/02/19), which was described in our previous article (Shen et al., 2021).

### Treatment and clinical assessment

The subjects were subjected to an 8-month intensive meditation-based intervention in conjunction with a comprehensive general rehabilitation program. The detailed experimental procedure, the therapeutic protocol of meditation has been previously described in the literature (21). All subjects completed the Positive and Negative Syndrome Scale (PANSS) (22, 23) and the Repeatable Battery for the Assessment of Neuropsychological Status (RBANS) (24, 25) to evaluate psychotic symptoms and cognitive function. PANSS scale is a 30-item scale used to evaluate the presence, absence and severity of Positive, Negative and General Psychopathology symptoms of schizophrenia. The 30 items are arranged as seven positive symptom subscale items, seven negative symptom subscale items, and 16 general psychopathology symptom items. RBANS scale is used to assess the cognitive function of adult patients with neurological dysfunction, such as dementia, head trauma and stroke. It is easy to operate and covers five cognitive domains: immediate memory, visuospatial structure, language, attention and delayed memory.

### MRI data acquisition and processing

MRI data were collected at three distinct time points: baseline, at the three-month mark, and again at the eight-month point during the treatment period. MRI data acquisition has also been described in previous studies (21).

Resting-state MRI preprocessing is based on the MATLAB (R2022a) data analysis platform and is completed via the SPM12 (www.fil.ion.ucl.ac.uk) tool kit DPARSF7.0 (26). The preprocessing steps include the following steps: (1) To avoid the impact of machine instability and other factors on data quality at the first few time points, the first 10 time points were removed, and the total number of data time points included in the subsequent calculation was 230. (2) Spatial layer correction is used to avoid impact on the image caused by head movement. (3) The EPI template was used to standardize the image to the Montreal Neurological Institute (MNI) space with resampling of 3 × 3 × 3 mm^3^ per voxel. (4) The wavelet denoising algorithm is used to remove signal peak points. (5) Regression covariates, including Friston-24 head motion parameters, white matter signals, cerebrospinal fluid signals, etc.; (6) filtering, retaining 0.01–0.08 Hz low-frequency signals (27). The data for the subsequent calculations were preprocessed.

### Functional connection analysis of the insula

According to the study by Deen et al., we subdivided the insula into six subregions: the left ventral anterior insula (L-vAI), right ventral anterior insula (R-vAI), left dorsal anterior insula (L-dAI), right dorsal anterior insula (R-dAI), left posterior insula (L-PI), and right posterior insula (R-PI) (28). These six regions were defined as the region of interest (ROI). For each region of interest (ROI), a representative time course was generated by averaging the time series within that ROI. Pearson correlation analysis was then performed to assess the relationship between this mean ROI signal and the time series of every voxel in the brain. This yielded functional connectivity maps between each insular subregion seed and all vertices across the whole brain surface for each participant. Finally, to create a group-level representation of functional connectivity, individual Fisher’s r-to-z transformed correlation maps were averaged within each group for each seed region.

### Statistical analysis

SPSS 26 (IBM Corporation, New Orchard Road, Armonk, NY 10504, USA) was used for examination of the demographic data pertaining to all the subjects. One-Way Repeated Measures ANOVA was employed to calculate the scores of the PANSS scales and RBANS scales for the patient group and to evaluate the interaction effect of time. A *post-hoc* analysis was performed afterward, and Bonferroni correction was used with p < 0.05 considered to indicate statistical significance.

The DPABI software package was used to perform an analysis of covariance (Two sample t-test) on the FC result of the healthy control and meditation group baseline, and Gaussian random field (GRF) correction was performed. The correction threshold with voxel p < 0.001 and cluster p < 0.05 was considered statistically significant. The mean FC values of the brain regions that were significantly different during the comparison were extracted as regions of interest (ROI). One-Way Repeated Measures ANOVA for three periods for each ROI in the meditation group, a *post-hoc* analysis was performed afterward, and least significant difference (LSD) correction was used with p < 0.05 considered to indicate statistical significance. For power analysis, we extracted the mean functional connectivity (FC) values from clusters showing significant group differences identified by the two-sample t-tests. Power analyses were then performed on these mean FC values for both the two-sample comparisons and the *post-hoc* paired t-tests to assess the adequacy of the current sample size in detecting the observed effects and to ensure the reliability of the statistical findings. The FC values of the brain regions with significant differences during the comparison were extracted and correlated with the scores of the clinical scales PANSS, RBANS.

## Results

### Demographic characteristics

Following an eight-month experimental process, 59 subjects completed all the requisite scale evaluations and MRI scans, after excessive fMRI head movement (> 2.5 mm) and missing follow-up data, finally including 26 in the meditation group and 29 in the healthy control group. All the subjects were male. Age had no significant differences between the two groups (*t*=1.40, *p* > 0.05) (**Table 1**).

**Table 1:** Demographic and clinical data at baseline.

	Schizophrenia (n=26)	Health control (n=29)	t	P value
Age (years)	57.73 ± 8.76	54.76 ± 6.63	1.40	0.17^a^
Education (years)	13.69 ± 0.74	/	/	
BMI (kg/ ${\text{m}}^{2})$	24.78 ± 3.22	/	/	
CPZ dose (mg/d)	388.0 ± 169.0	/	/	
PANSS TScore	91.23 ± 14.49	/	/	
RBANS Tscores	73.58 ± 13.64	/	/	

^a^Two independent-samples t-test (26 patients vs 29 health control); PANSS, Positive and Negative Symptoms Scale; T, total; RBANS, Repeatable Battery for the Assessment of Neuropsychological Status; CPZ dose, chlorpromazine dose.

### Effect of meditation treatment on clinical symptoms

The RMANOVA analysis revealed significant time effects for both the PANSS total score and its subscale scores. *Post-hoc* analysis demonstrated that, compared to baseline scores, the total and subscale scores of the PANSS showed a consistent reduction at both the three-month and eight-month follow-ups. *Post-hoc* analysis revealed that there were no significant differences of RBNAS scores in the third and eighth months compared with the baseline (**Table 2**).

**Table 2:** The PANSS scale scores at different time points.

	Baseline	Third month	Eighth month	F(p)
PANSS PScore	27.46 ± 5.31	23.46 ± 4.75	19.88 ± 4.88	69.63 (<0.001)
PANSS NScore	22.61 ± 6.91	21.26 ± 6.07	20.50 ± 6.34	22.45 (<0.001)
PANSS GScore	41.15 ± 7.75	38.61 ± 6.80	35.85 ± 7.28	36.71 (<0.001)
PANSS TScore	91.23 ± 14.49	83.35 ± 12.26	76.23 ± 13.96	77.99 (<0.001)

PANSS, Positive and Negative Symptoms Scale (P, positive; N, negative; G, general; T, total).

### Effect of meditation treatment on brain functional connectivity

#### Insula subregion: L-dAI whole-brain functional connectivity results

**Figure 1:**
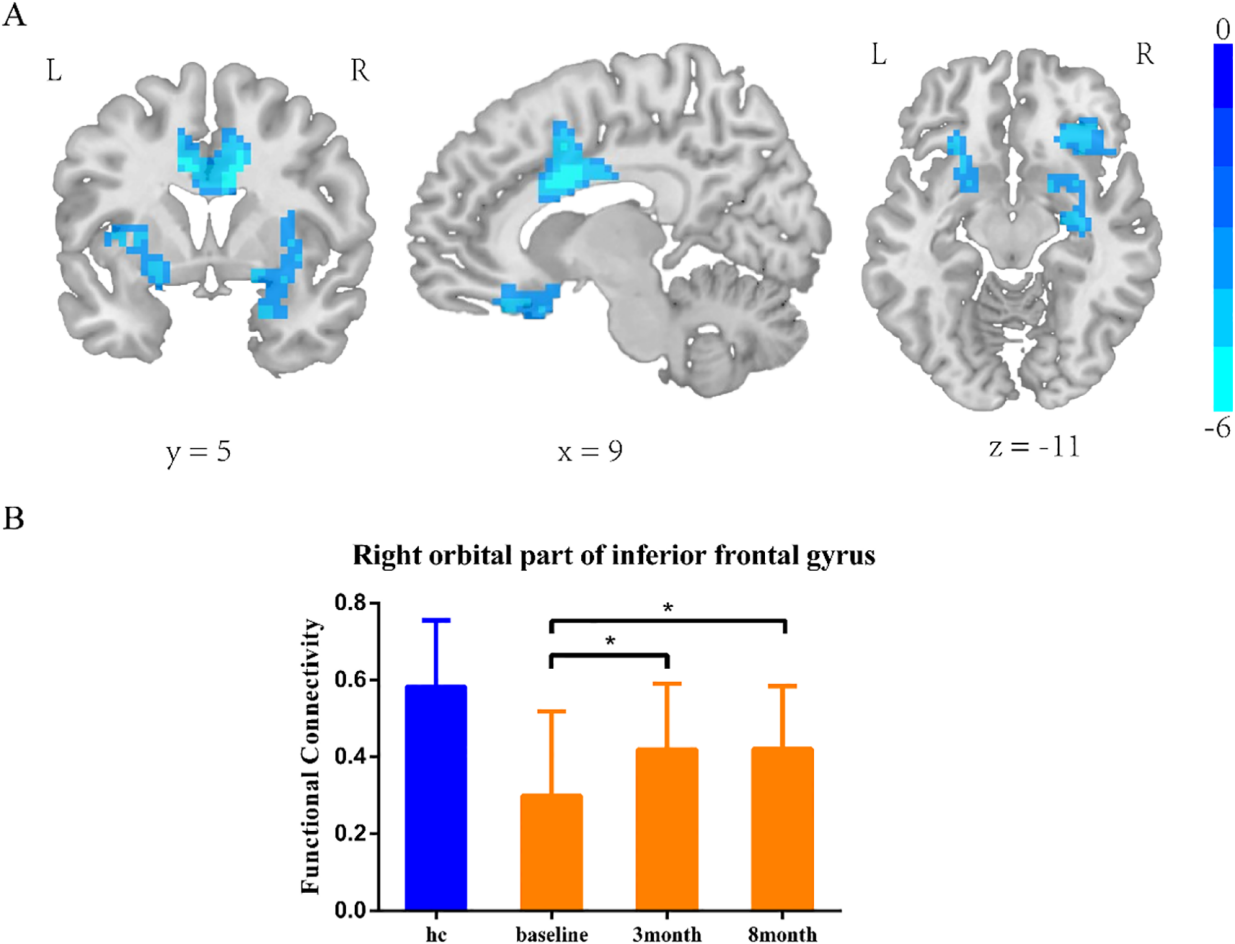
L-dAI two-sample t-test results. **(A)** SZ group showed reduced functional connectivity in right orbital inferior frontal gyrus, left lentiform nucleus, and median cingulate-paracingulate regions at baseline. **(B)** Significant time effect was observed in right orbital inferior frontal gyrus connectivity. (one-way ANOVA results. *LSD corrected, *p* < 0.05).

The two-sample t-test results showed significant differences after correction (GRF correction, voxel *p* < 0.001, cluster *p* < 0.05, coordinate showed in **Table 3**) in the right orbital part of the inferior frontal gyrus, left lentiform nucleus, and right median cingulate and paracingulate, as shown in **Figure 1A**. A one-way ANOVA of these three ROIs found a significant time difference in the right orbital part of inferior frontal gyrus (*F* = 3.651, *p* = 0.031, **Figure 1B**). *Post hoc* analyses showed significant improvement of functional connectivity in the meditation group at the third and eighth month compared with baseline (LSD correction, *p* < 0.05; LSD correction, *p* < 0.05).

**Table 3:** Coordinate of significant regions in L-dAI two sample t-test results.

Regions	MNI			Voxel size	T value
	X	Y	Z		
Right orbital part of inferior frontal gyrus	30	30	-9	576	-5.0311
Left lentiform nucleus	-27	24	-12	206	-4.9083
Median cingulate and paracingulate	9	9	30	336	-6.0942

#### Insula subregion: L-dAI correlation analysis results

PANSS and RBANS were not found to be associated with the FC of the L-dAI connectivity anomaly ROI.

#### Insula subregion: L-PI whole-brain FC results

**Figure 2:**
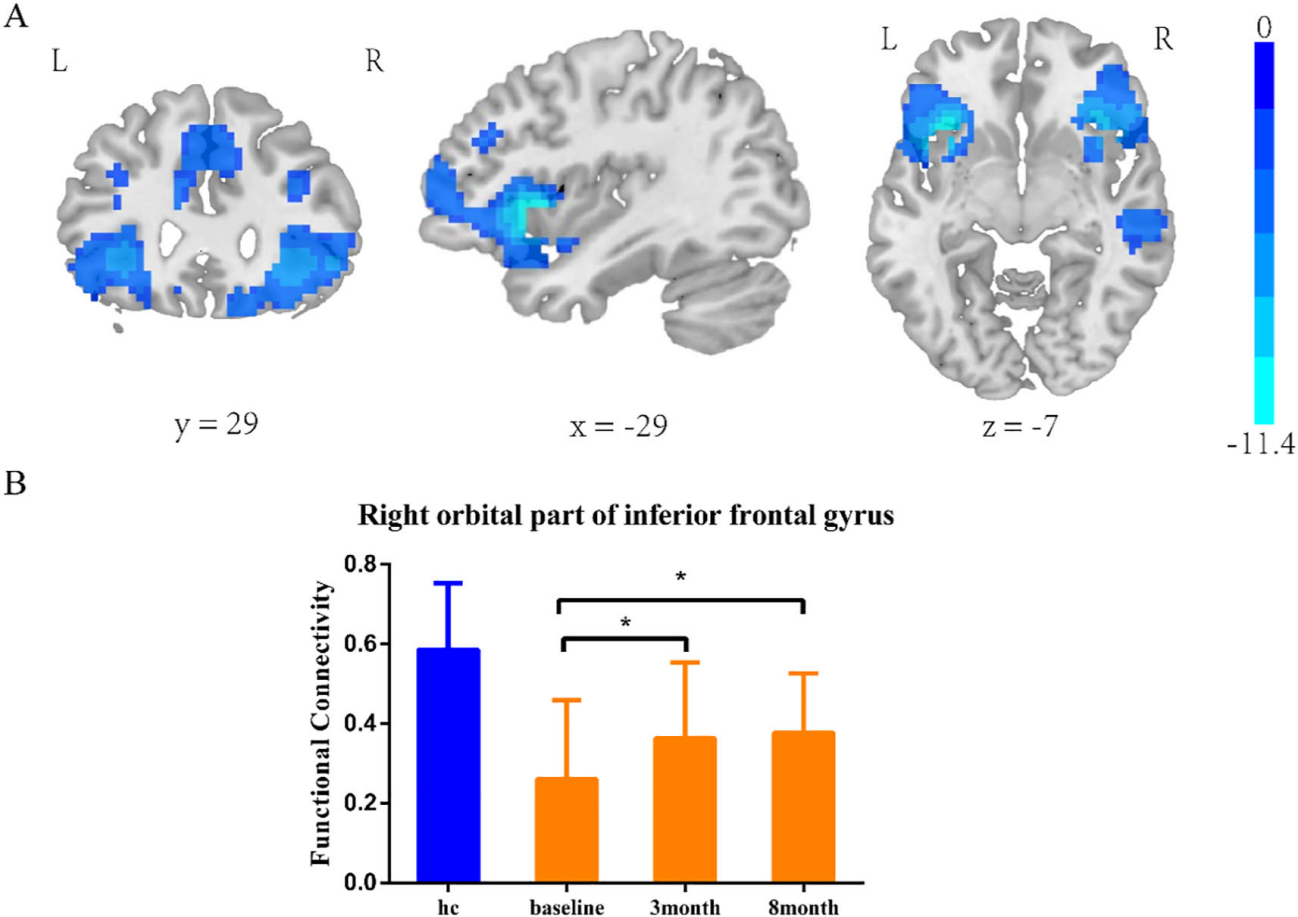
L-PI two-sample t-test results. e **(A)** SZ group showed reduced functional connectivity in right orbital inferior frontal gyrus, left orbital part of inferior frontal gyrus, middle temporal gyrus and median cingulate-paracingulate regions at baseline. **(B)** Significant time effect was observed in right orbital inferior frontal gyrus connectivity. (one-way ANOVA results. *LSD corrected, *p* < 0.05).

The two-sample t-test results showed significant differences after correction (GRF correction, voxel *p* < 0.001, cluster *p* < 0.05, coordinate showed in **Table 4**) in the right orbital part of inferior frontal gyrus, left orbital part of inferior frontal gyrus, right middle temporal gyrus and right median cingulate and paracingulate, as shown in **Figure 2A**. A one-way ANOVA of these three ROIs found a significant time difference in the right orbital part of inferior frontal gyrus (*F* = 3.193, *p* = 0.047, **Figure 2B**). *Post hoc* analyses showed significant improvement of functional connectivity in the meditation group at the third and eighth month compared with baseline (LSD correction, *p* < 0.05; LSD correction, *p* < 0.05).

**Table 4:** Coordinate of significant regions in L-PI two sample t-test results.

Regions	MNI			Voxel size	*T* value
	X	Y	Z		
Right orbital part of inferior frontal gyrus	39	21	-6	988	-7.8618
Left orbital part of inferior frontal gyrus	-36	21	-6	1288	-11.3772
Middle temporal gyrus	51	-21	-6	148	-4.7847
Median cingulate and paracingulate	9	15	27	948	-6.3572

#### Insula subregion: L-PI correlation analysis results

PANSS and RBANS were not found to be associated with the FC of the L-PI connectivity anomaly ROI.

#### Insula subregion: L-vAI whole-brain FC results

**Figure 3:**
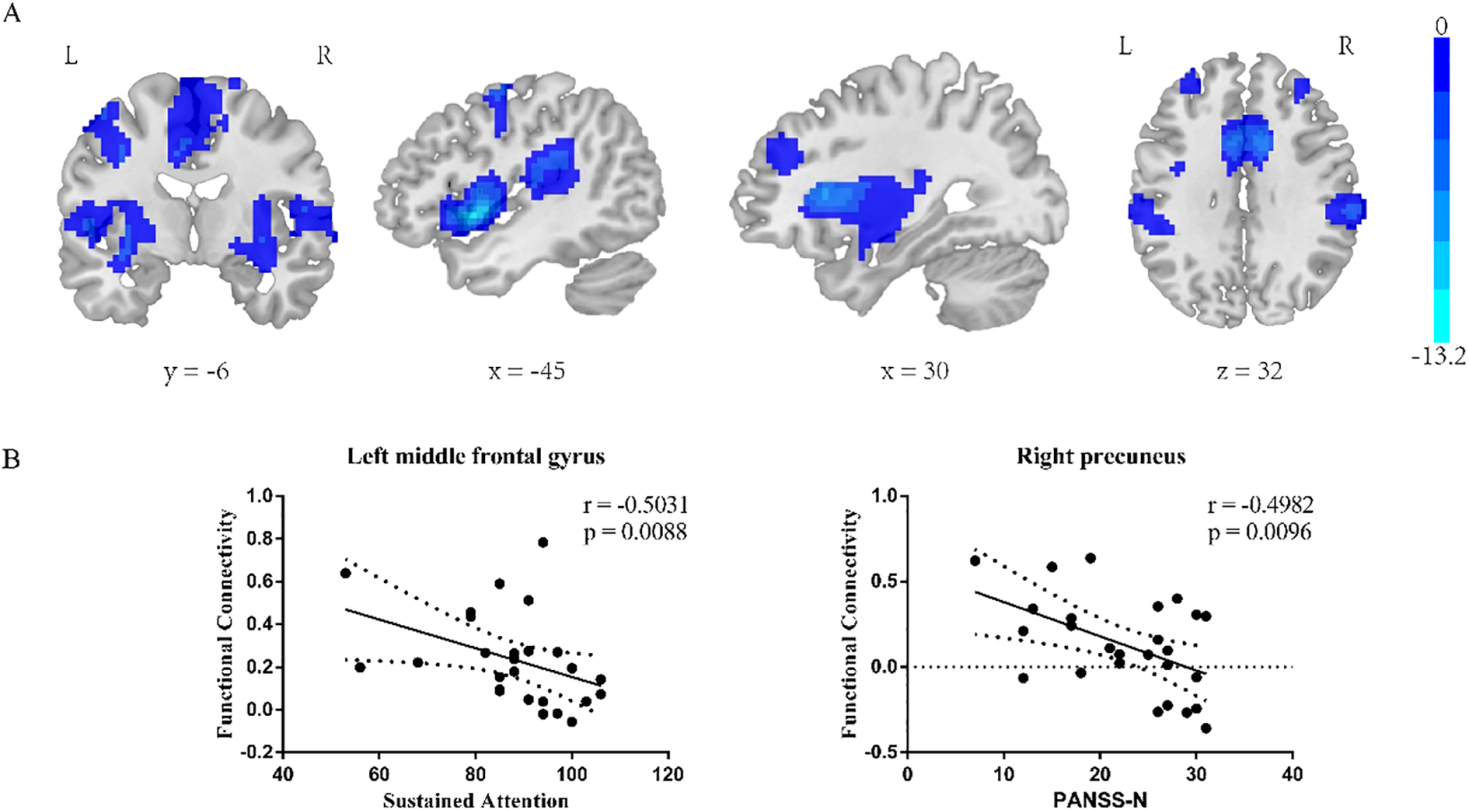
L-vAI two-sample t-test results **(A)**. Regions exhibiting significantly lower functional connectivity in the SZ group compared to the HC group. GRF corrected, voxel *p* < 0.001, cluster *p* < 0.05. **(B)** left: correlation analysis between the RBANS sustained attention score and left middle frontal gyrus FC value in L-vAI, right: correlation analysis between the PANSS Nscore and right cuneus FC value in L-vAI.

The two-sample t-test results showed significant differences after correction (GRF correction, voxel *p* < 0.001, cluster *p* < 0.05, coordinate showed in **Table 5**) in the right orbital part of inferior frontal gyrus, left orbital part of inferior frontal gyrus, right middle temporal gyrus, right median cingulate, left precentral gyrus and right cuneus as shown in **Figure 3A**. A one-way ANOVA of these six ROIs found no significant within-group effects.

**Table 5:** Coordinate of significant regions in L-vAI two sample t-test results.

Regions	MNI			Voxel size	*T* value
	X	Y	Z		
Right orbital part of inferior frontal gyrus	-39	15	3	1822	-13.2279
Left orbital part of inferior frontal gyrus	33	12	6	2055	-10.2567
Left middle temporal gyrus	-33	48	27	179	-7.0003
Median cingulate and paracingulate	6	12	42	1326	-8.5784
Left precentral gyrus	-45	-3	57	132	-6.0684
Right cuneus	-12	-51	60	147	-4.367

#### Insula subregion: L-vAI correlation analysis results

The L-vAI-left middle frontal gyrus FC value in schizophrenia patients was negative correlated with the RBANS sustained attention score (*p* = 0.0088, *r* = -0.5031), the L-vAI-right cuneus FC value in schizophrenia patients was negative correlated with the PANSS Nscore (*p* = 0.0096, *r* = -0.4982, scatter plot was shown in **Figure 3B**).

#### Insula subregion: R-dAI whole-brain FC results

**Figure 4:**
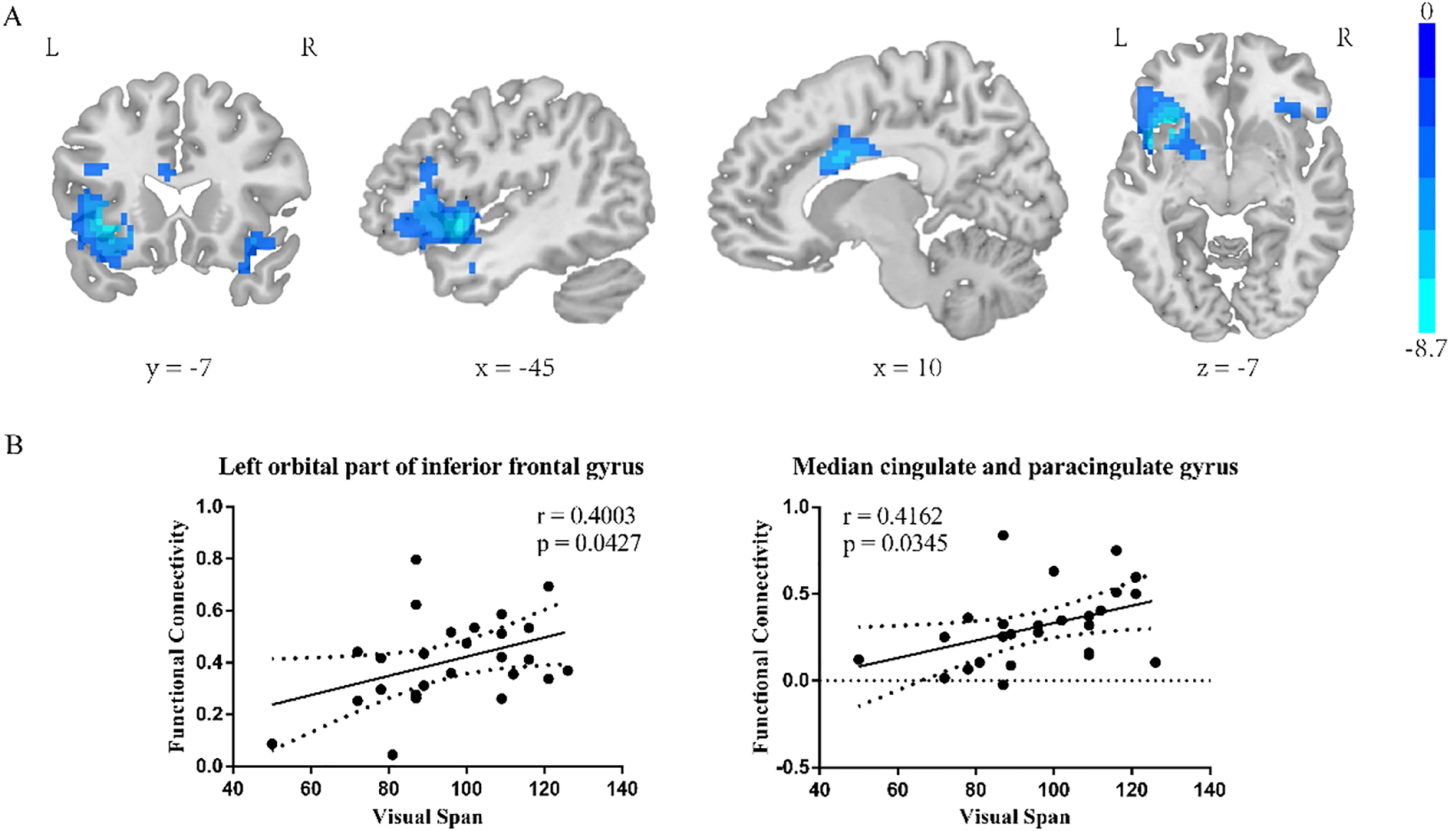
R-dAI two-sample t-test results. **(A)** SZ group showed reduced functional connectivity in right orbital inferior frontal gyrus, left orbital part of inferior frontal gyrus, middle temporal gyrus and median cingulate-paracingulate regions at baseline. GRF corrected, voxel *p* < 0.001, cluster *p* < 0.05. **(B)** Correlation of R-dAI- left orbital part of inferior frontal gyrus FC value and R-dAI- median cingulate and paracingulate gyrus FC value on RBANS Visual span score.

The two-sample t-test results showed significant differences after correction (GRF correction, voxel *p* < 0.001, cluster *p* < 0.05, coordinate showed in **Table 6**) in the right orbital part of inferior frontal gyrus, left orbital part of inferior frontal gyrus and median cingulate and paracingulate gyrus as shown in **Figure 4A**, one-way ANOVA of these six ROIs found no significant within-group effects.

**Table 6:** Coordinate of significant regions in R-dAI two sample t-test results.

Regions	MNI			Voxel size	*T* value
	X	Y	Z		
Right orbital part of inferior frontal gyrus	27	27	-9	151	-4.8533
Left orbital part of inferior frontal gyrus	-33	12	-3	702	-8.6972
Median cingulate and paracingulate gyrus	9	12	27	307	-6.2911

#### Insula subregion: R-dAI correlation analysis results

The R-dAI- left orbital part of inferior frontal gyrus and the R-dAI- median cingulate and paracingulate gyrus FC value in schizophrenia patients was positive correlated with the RBANS Visual Span score (*p*=0.0427, *r*=0.4003; *p*=0.0345, *r*=0.4162, scatter plot was shown in **Figure 4B**).

#### Insula subregion: R-PI whole-brain FC results

**Figure 5:**
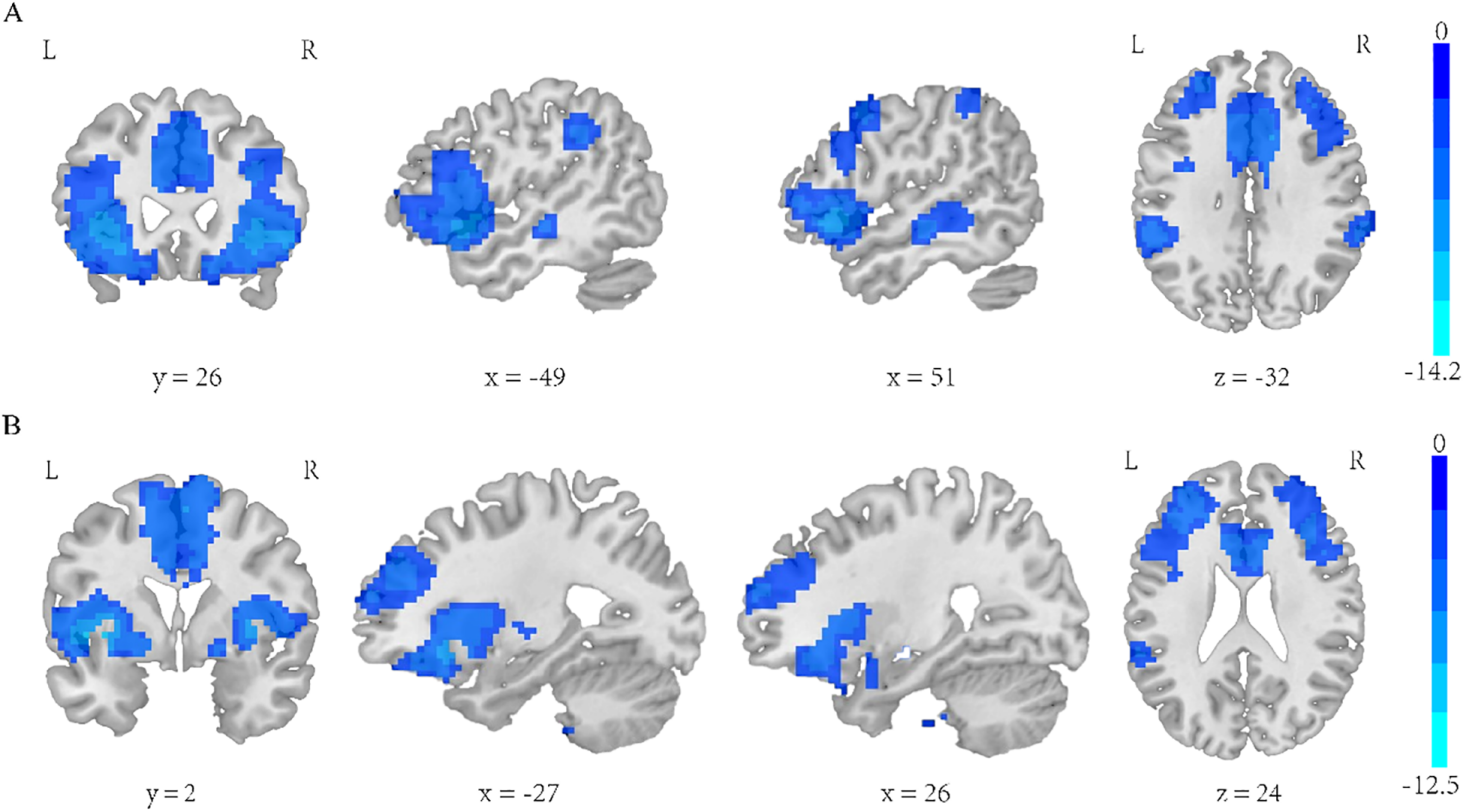
**(A)** Regions exhibiting significantly lower functional connectivity within the R-PI network in the SZ group compared to the HC group. **(B)** Regions exhibiting significantly lower functional connectivity within the R-vAI network in the SZ group compared to the HC group. GRF corrected, voxel *p* < 0.001, cluster *p* < 0.05.

The two-sample t-test results showed significant differences after correction (GRF correction, voxel *p* < 0.001, cluster *p* < 0.05, coordinate showed in **Table 7**) in the right orbital part of inferior frontal gyrus, left orbital part of inferior frontal gyrus, middle frontal gyrus, left supramarginal, median cingulate and paracingulate gyrus and right supramarginal gyrus as shown in **Figure 5A**. A one-way ANOVA of these six ROIs found no significant within-group effects.

**Table 7:** Coordinate of significant regions in R-PI two sample t-test results.

Regions	MNI			Voxel size	*T* value
	X	Y	Z		
Right orbital part of inferior frontal gyrus	51	24	-3	2250	-9.0591
Left orbital part of inferior frontal gyrus	-39	15	3	2492	-14.2011
Middle frontal gyrus	54	-21	-9	233	-5.7272
Left supramarginal gyrus	-54	-42	30	318	-6.0878
Median cingulate and paracingulate	9	15	27	1512	-7.9774
Right supramarginal gyrus	63	-36	45	226	-5.6503

#### Insula subregion: R-PI correlation analysis results

PANSS and RBANS scores were not found to be associated with the FC of the R-PI connectivity anomaly ROI.

#### Insula subregion: R-vAI whole-brain FC results

The two-sample t-test results showed significant differences after correction (GRF correction, voxel *p* < 0.001, cluster *p* < 0.05, coordinate showed in **Table 8**) in the right orbital part of inferior frontal gyrus, left orbital part of inferior frontal gyrus and median cingulate and paracingulate gyrus as shown in **Figure 5B**. A one-way ANOVA of these six ROIs found no significant within-group effects.

**Table 8:** Coordinate of significant regions in R-vAI two sample t-test results.

Regions	MNI			Voxel size	*T* value
	X	Y	Z		
Right orbital part of inferior frontal gyrus	33	12	6	841	-8.9758
Left orbital part of inferior frontal gyrus	-33	12	-3	1636	-12.5248
Median cingulate and paracingulate	9	9	45	1313	-7.0491

#### Insula subregion: R-vAI correlation analysis results

PANSS and RBANS scores were not found to be associated with the FC of the R-vAI connectivity anomaly ROI.

Given the relatively limited sample size in this study, to enhance the reliability of the results, we conducted detailed power analyses as a supplement to all statistical analyses presented in the main text. The tables list the mean ± standard deviation for each brain region, mean differences with their 95% confidence intervals, Cohen’s d effect sizes, and statistical power values. Power analysis evaluates the sensitivity and statistical efficacy of the current sample size in detecting true effects.(For details, please refer to the **Supplementary Materials**).

## Discussion

This 8-month prospective study revealed that there were significant differences between the fMRI findings in the meditation group and those in the HC group at baseline. Compared with HC group, the dynamic functional connectivity in the left orbitofrontal gyrus, right orbitofrontal gyrus, medial and paracingulate gyrus, right hippocampus, and left auxiliary motor area in the meditation group was significantly reduced. At the third and eighth month, schizophrenia patients in the meditation group showed a significant improvement of functional connectivity between L-dAI、L-PI and right orbital part of inferior frontal gyrus compared with baseline. Similar to the results of a longitudinal study, this study revealed that at the eighth month, the total PANSS score and the scores of each factor of patients in the meditation group decreased significantly from baseline (19), but we did not find any significant improvement in the total score of cognitive function or the scores of each factor in the meditation group. In 1995, the Dysconnection Hypothesis was proposed for schizophrenia (29). Subsequently, an increasing number of studies have confirmed that brain dysconnection may be a possible pathological mechanism of schizophrenia (13, 30, 31). The relationship was confirmed in recent work between right middle frontal gyrus regional homogeneity values and general PANSS scores in schizophrenia (32). Research indicates a link between 5 specific subnetworks based on functional connectivity centered on the dorsolateral superior frontal gyrus, orbital part of inferior frontal gyrus, superior occipital gyrus, hippocampus, and parahippocampal gyrus and clinical variables within the schizophrenia patients, specifically, the right orbital part of the inferior frontal gyrus, the left superior occipital gyrus, and the left hippocampus subnetworks, showed predictive power for PANSS paranoid/belligerence scores (33). Our study showed the dysconnection between insula subregion and the left orbitofrontal gyrus, right orbitofrontal gyrus, medial and paracingulate gyrus, right hippocampus, and left auxiliary motor area and further confirmed that schizophrenia patients in the meditation group showed significant improvement of functional connectivity between L-dAI、L-PI and right orbital part of inferior frontal gyrus at the third and eighth month.

### Abnormal functional connectivity of insula in patients with schizophrenia

This study revealed that the functional connections between the insula and the left orbitofrontal gyrus, the right orbitofrontal gyrus, the medial and paracingulate gyri, the right hippocampus, and the left auxiliary motor area in schizophrenia patients were significantly reduced compared with those in HCs. The present study corroborates the findings of preceding research on the subject of insular connectivity dysfunction. A study revealed that decreased activity in the right anterior insula salient network(SN) was associated with increased functional connectivity between hallucinations and the default mode network and the central executive network(DMN/CEN) in patients with schizophrenia, providing evidence for an abnormal dependence of DMN/CEN interactions on the activity of the anterior insula salient network and correlating impaired insula, DMN, and CEN activity with the psychotic symptoms of schizophrenia (34). Evidence suggested the volume and thickness of the insula in patients with schizophrenia were associated with cognitive function and both positive and negative symptoms (35). Study revealed that the functional connection between the right anterior insula and CEN、DMN was significantly enhanced in patients with schizophrenia when they performed tasks, and the influence of the insula on the CEN was more obvious in patients with a greater burden of negative symptoms (36). Research indicated that excitatory functional connections from the dorsal anterior cingulate cortex to the anterior insula were observed in schizophrenia patients (37). One study used genome-wide association analysis (GWAS) to calculate the correlation of polygenic risk scores (PRSs) in a cohort of adults with schizophrenia in the UK Biobanks with their gray and white matter microstructures and reported that the brain regions associated with the genetic mechanisms of schizophrenia include the temporal lobe, cingulate and prefrontal cortex regions, insula and hippocampus (38). In conclusion, many studies have reported the relationship between abnormal dynamic functional connectivity of the insula and schizophrenia, especially the close connection between abnormal dynamic functional connectivity between the insula and DMN/CEN/SN and schizophrenia.

### The efficacy of meditation on insula

Three prominent, functionally connected large-scale networks, namely, the default mode network (DMN), central execution network (CEN) and Salience Network (SN), are collectively referred to as triple networks. These three networks play crucial roles in brain diseases, as well as in fundamental neuroscience processes such as mindfulness (39, 40). One study reported that the major nodes of the DMN were relatively inactivated in experienced meditators, and functional connectivity analysis revealed that coupling between the posterior cingulate, dorsal anterior cingulate, and dorsolateral prefrontal cortex was stronger at baseline and during meditation (41). A longitudinal study revealed changes in brain function after a mindfulness-based intervention, which involved brain activity and functional connections in the Default Mode Network, Central Execution Network and Salience Network (42). Most recent neuroimaging studies on mindfulness have focused on changes in functional connections (FCs) within and between the triple network (43, 44). In conclusion, mindfulness meditation can activate the brain’s neuroplasticity and improve the functional connectivity of local brain areas, especially in the DMN, CEN and SN. Studies showed that American soldiers trained in meditation were able to alter brain activation (reducing activation of the right insula and anterior cingulate gyrus), allowing individuals to process aversive interoceptive stimuli more effectively (45, 46). A meta-analysis revealed that mindfulness meditation increased the volume of gray matter in subjects’ right anterior abdominal insula, and another meta-analysis revealed that the brain regions that sustained changes during long-term meditation practice included the insula and other regions that affected interoceptive somatic awareness (47, 48). The relationship was confirmed in recent work that long-term meditation practitioners increased the thickness of the left ventrolateral prefrontal cortex and the anterior insula cortex (49). Another study revealed that meditation practitioners presented significantly greater functional connectivity related to the insula in the thalamus, caudate nucleus, middle frontal gyrus, and superior temporal gyrus (50). As part of the SN, the role of the insula in the meditation-schizophrenia relationship is unclear because of a lack of relevant research.

Many previous studies have confirmed that meditation intervention can effectively improve the clinical symptoms and prognosis of patients with schizophrenia, but the main outcome indicators are mostly limited to the clinical symptom scale. The brain imaging, especially whole-brain functional connectivity with the insula as the seed, is rarely used as a measurement index (51–53). Researchers reported that increased functional connectivity between the left anterior insula and the right anterior middle cingulate cortex was negatively correlated with emotion management in schizophrenia patients (54). Previous studies have shown that meditation practices can effectively improve the symptoms and prognosis of patients with schizophrenia, and the mechanism may involve an increase in cortical thickness in brain regions such as the insula and changes in dynamic functional connectivity. The abnormal functional connectivity of the insula may be pivotal in the manifestation of the functions and symptoms of schizophrenia. Consequently, this area should be the focus of special attention. Therefore, this study further explored the effects of meditation interventions on the dynamic functional connectivity of the whole brain seeded by different subregions of the insula in patients with schizophrenia in a prospective study of meditation practice for 8 months. Even though the subjects of our intervention are chronic schizophrenia patients whose functional connections in the brain are in a relatively stable decreased state, we found that there was a significant improvement of functional connectivity between L-dAI、L-PI and right orbital part of inferior frontal gyrus at the third and eighth month compared with baseline. This may be the neuroimaging mechanism by which meditation interferes with schizophrenia.

## Limitations

There are several limitations in this study. First, the sample size of this study was small, and the final effective sample of the meditation group included 26 patients with chronic schizophrenia and 29 subjects in the HC group. A small sample size may lead to overfitting of the statistical model and affect the final result. Second, the subjects of this study were mainly Han people in Shanghai, and the results obtained are difficult to extend to other regions and populations. Third, owing to the small sample size, further subgroup analysis could not be conducted, such as for patients with mainly negative symptoms/mainly positive symptoms, and the correlation between the functional connectivity of various brain regions and specific psychotic symptoms of subjects could not be further explored. Fourth, this study was lack of a control group of schizophrenia patients on conventional drug therapy. This is not a strictly controlled study, but rather an attempt to clarify the neuroimaging mechanism of meditation intervention in schizophrenia through before-and-after comparisons within the meditation group itself, the control group is used to identify the differential brain regions between schizophrenia patients and normal people. Despite existing the confounding effects of medication, we selected patients with chronic stable schizophrenia who remained unchanged antipsychotic medication during eight months of meditation intervention, somewhat mitigating the effect of medication on the final outcome. In future study we will include a control group with conventional treatment.

## Conclusion

This 8-month study demonstrated that meditation significantly enhanced the functional connectivity of the entire brain with the insula subregion in patients diagnosed with schizophrenia, particularly in relation to overall and positive psychotic symptoms. These findings suggest that the insula may serve as a distinctive imaging marker and a viable intervention target for both meditation in the treatment of schizophrenia.

## Data Availability

The raw data supporting the conclusions of this article will be made available by the authors, without undue reservation.
