# Supplementary material for: The efficacy of meditation on insula dysfunction in patients with chronic schizophrenia
Source: Front Psychiatry. 2025 Sep 2;16:1622594. doi: 10.3389/fpsyt.2025.1622594 (PMC12436383; doi:10.3389/fpsyt.2025.1622594)
Supplement: Supplementary file 1 [file Table1.docx]

**Supplementary Materials**

**Title:**

**The efficacy of meditation on insula dysfunction in patients with chronic schizophrenia**

The file includes: Table S1-S3

Given the relatively limited sample size in this study, to enhance the reliability of the results, we conducted detailed power analyses as a supplement to all statistical analyses presented in the main text. The tables list the mean ± standard deviation for each brain region, mean differences with their 95% confidence intervals, Cohen’s d effect sizes, and statistical power values. Power analysis evaluates the sensitivity and statistical efficacy of the current sample size in detecting true effects. Specifically:

Table S1 presents the power analysis of two-sample t-tests comparing functional connectivity between healthy controls (HC) and patients at baseline. The Cohen's d effect sizes across brain regions ranged from 1.00 to 1.99, with statistical power consistently high, between 0.9 and 1.0, indicating that the current sample size is sufficient to reliably detect significant group differences.

Table S2 shows the power analysis for paired t-tests comparing patients before and after 3 months of treatment. The observed Cohen's d values were 0.51 and 0.41, with corresponding power values of 0.7 and 0.519, reflecting moderate effect sizes and somewhat limited power, suggesting lower statistical sensitivity for detecting short-term treatment effects.

Table S3 presents the power analysis of paired t-tests comparing patients before and after 8 months of treatment. Cohen's d ranged from 0.54 to 0.57, and power increased to between 0.757 and 0.801, indicating an enhanced detection capability for longer-term treatment effects. The power values approach or meet the commonly accepted threshold of 0.8, suggesting that the sample size is relatively adequate for detecting treatment effects during this period.

Overall, the power analysis results support the robustness of the study's conclusions, while also highlighting the need to consider statistical power limitations when interpreting treatment-related changes.

Table S1. Power analysis of two-sample t-tests: HC vs. baseline patients.

| ROI | Regions | HC(Mean±SD) | Baseline (Mean±SD) | Mean_diff | 95% CI | Cohen d | Power |
| --- | --- | --- | --- | --- | --- | --- | --- |
| L-dAI | Right orbital part of inferior frontal gyrus | 0.58±0.17 | 0.30±0.22 | 0.28 | 0.18~0.39 | 1.45 | 1.000 |
|  | Left lentiform nucleus | 0.69±0.22 | 0.42±0.20 | 0.27 | 0.15~0.38 | 1.27 | 0.991 |
|  | Median cingulate and paracingulate | 0.88±0.25 | 0.31±0.32 | 0.57 | 0.41~0.73 | 1.99 | 1.000 |
| L-PI | Right orbital part of inferior frontal gyrus | 0.59±0.17 | 0.26±0.20 | 0.33 | 0.23~0.43 | 1.79 | 0.999 |
|  | Left orbital part of inferior frontal gyrus | 0.60±0.16 | 0.28±0.18 | 0.32 | 0.23~0.41 | 1.88 | 0.999 |
|  | Middle temporal gyrus | 0.33±0.16 | 0.09±0.30 | 0.24 | 0.11~0.38 | 1.02 | 0.999 |
|  | Median cingulate and paracingulate | 0.65±0.19 | 0.28±0.30 | 0.36 | 0.23~0.49 | 1.55 | 0.999 |
| L-vAI | Right orbital part of inferior frontal gyrus | 0.61±0.18 | 0.26±0.20 | 0.34 | 0.24~0.45 | 1.80 | 1.000 |
|  | Left orbital part of inferior frontal gyrus | 0.66±0.17 | 0.30±0.20 | 0.35 | 0.25~0.45 | 1.94 | 1 |
|  | Left middle temporal gyrus | 0.53±0.18 | 0.23±0.22 | 0.30 | 0.19~0.41 | 1.51 | 0.999 |
|  | Median cingulate and paracingulate | 0.68±0.17 | 0.30±0.24 | 0.38 | 0.27~0.50 | 1.87 | 1.000 |
|  | Left precentral gyrus | 0.45±0.13 | 0.20±0.22 | 0.25 | 0.15~0.35 | 1.38 | 0.999 |
|  | Right cuneus | 0.40±0.16 | 0.13±0.28 | 0.27 | 0.15~0.40 | 1.22 | 0.999 |
| R-dAI | Right orbital part of inferior frontal gyrus | 0.57±0.21 | 0.31±0.25 | 0.26 | 0.14~0.39 | 1.14 | 0.991 |
|  | Left orbital part of inferior frontal gyrus | 0.72±0.18 | 0.41±0.17 | 0.31 | 0.21~0.40 | 1.76 | 1.000 |
|  | Median cingulate and paracingulate gyrus | 0.66±0.20 | 0.31±0.22 | 35 | 0.23~0.46 | 1.65 | 0.999 |
| R-PI | Right orbital part of inferior frontal gyrus | 0.51±0.17 | 0.18±0.20 | 0.33 | 0.23~0.43 | 1.77 | 0.999 |
|  | Left orbital part of inferior frontal gyrus | 0.54±0.16 | 0.20±0.18 | 0.34 | 0.24~0.43 | 1.98 | 1 |
|  | Middle frontal gyrus | 0.31±0.16 | 0.06±0.25 | 0.25 | 0.14~0.37 | 1.22 | 0.999 |
|  | Left supramarginal gyrus | 0.46±0.17 | 0.17±0.22 | 0.28 | 0.18~0.39 | 1.46 | 0.999 |
|  | Median cingulate and paracingulate | 0.64±0.18 | 0.25±0.25 | 0.38 | 0.27~0.50 | 1.80 | 1.000 |
|  | Right supramarginal gyrus | 0.42±0.16 | 0.20±0.21 | 0.22 | 0.11~0.32 | 1.15 | 0.996 |
| R-vAI | Right orbital part of inferior frontal gyrus | 0.70±0.20 | 0.35±0.20 | 0.34 | 0.24~0.45 | 1.75 | 0.999 |
|  | Left orbital part of inferior frontal gyrus | 0.68±0.17 | 0.31±0.20 | 0.36 | 0.26~0.46 | 1.96 | 1.000 |
|  | Median cingulate and paracingulate | 0.67±0.17 | 0.31±0.23 | 0.36 | 0.25~0.47 | 1.81 | 1.000 |

Note：L-dAI :left ventral anterior insula; L-PI: left posterior insula; L-vAI: left ventral anterior insula; R- dAI: right dorsal anterior insula; R-PI: right posterior insula; R-vAI: right ventral anterior insula; Mean_diff : mean differences; CI: confidence intervals.

Table S2. Power analysis of paired t-tests: baseline vs. 3-month treatment.

| ROI | Regions | Baseline (Mean±SD) | 3month (Mean±SD) | Mean_diff | 95% CI | Cohen d | Power |
| --- | --- | --- | --- | --- | --- | --- | --- |
| L-dAI | Right orbital part of inferior frontal gyrus | 0.30±0.22 | 0.42±0.17 | 0.12 | 0.01~0.23 | 0.51 | 0.700 |
| L-PI | Right orbital part of inferior frontal gyrus | 0.26±0.20 | 0.36±0.19 | 0.10 | -0.01~0.21 | 0.41 | 0.519 |

Note：L-dAI :left ventral anterior insula; L-PI: left posterior insula; Mean_diff : mean differences; CI: confidence intervals.

Table S3. Power analysis of paired t-tests: baseline vs. 8-month treatment.

| ROI | Regions | Baseline (Mean±SD) | 8month (Mean±SD) | Mean_diff | 95% CI | Cohen d | Power |
| --- | --- | --- | --- | --- | --- | --- | --- |
| L-dAI | Right orbital part of inferior frontal gyrus | 0.30±0.22 | 0.42±0.16 | 0.12 | 0.01~0.23 | 0.57 | 0.801 |
| L-PI | Right orbital part of inferior frontal gyrus | 0.26±0.20 | 0.38±0.15 | 0.12 | 0.02~0.21 | 0.54 | 0.757 |

Note：L-dAI :left ventral anterior insula; L-PI: left posterior insula; Mean_diff : mean differences; CI: confidence intervals.
